# Evaluation of the Antimicrobial Effects of Olive Mill Wastewater Extract Against Food Spoiling/Poisoning, Fish-Pathogenic and Non-Pathogenic Microorganisms

**DOI:** 10.3390/microorganisms12112216

**Published:** 2024-10-31

**Authors:** Dilek Kahraman Yılmaz, Fevziye Işıl Kesbiç, Ekrem Şanver Çelik, Deniz Anıl Odabaşı, Sevdan Yilmaz, Hany M. R. Abdel-Latif

**Affiliations:** 1 Department of Marine and Inland Water Sciences, Faculty of Marine Sciences and Technology, Çanakkale Onsekiz Mart University, Çanakkale 17100, Türkiye; sanver_celik@comu.edu.tr (E.Ş.Ç.); aodabasi@comu.edu.tr (D.A.O.); 2 Central Research Laboratory, Kastamonu University, Kastamonu 37150, Türkiye; ikesbic@kastamonu.edu.tr; 3 Department of Aquaculture, Faculty of Marine Sciences and Technology, Çanakkale Onsekiz Mart University, Çanakkale 17100, Türkiye; sevdanyilmaz@comu.edu.tr; 4 Department of Poultry and Fish Diseases, Faculty of Veterinary Medicine, Alexandria University, Alexandria 22758, Egypt

**Keywords:** food control, aquaculture, disease, olive, alternative additives, fish pathogens

## Abstract

Although antibiotics are the main therapy for bacterial infections, the reports showed that the overuse (or misuse) of antibiotics will results in several problems such as the development of antibiotic-resistant strains, persistence of drug residues, and numerous environmental concerns. Therefore, finding antibiotic alternatives is considered of vital importance. Investigation of the antimicrobial properties of several plant substances and extracts is of great value to replace antibiotics. With this objective, this study aimed to evaluate the antimicrobial activities of an ethanolic extract prepared from olive mill wastewater (OMWW), which is a by-product of olive oil production with considerable environmental burden, against 38 bacterial strains, including fish-associated pathogens, non-pathogenic isolates, collection strains, and one yeast strain, *Candida albicans*. Disk diffusion, minimum inhibitory concentration (MIC), and minimum bactericidal/fungicidal concentration (MBC/MFC) tests were used to determine the antimicrobial activity of the OMWWE. According to the results, OMWWE provoked strong inhibitory effects against *Shewanella baltica* strain SY-S145. It also showed a moderate inhibitory effect on *Plesiomonas shigelloides* strain SY-PS16 and *Vibrio anguillarum* strain SY-L24. The MIC and MBC of OMWWE on *Shewanella baltica* SY-S145, *Vibrio gigantis* strain C24, and *V. anguillarum* strain SY-L24 were 500 µg/mL. The MIC and MBC on *V. parahaemolyticus* ATCC 17802 were 1000 µg/mL, whereas the values for *Aeromonas salmonicida* ATCC 33658 were 500 µg/mL and 1000 µg/mL, respectively. To put it briefly, the OMWW extract showed high antimicrobial activity and can act as an environmentally friendly additive for the control and prevention of diseases caused by *A. veronii*, *A. hydrophila*, *P. shigelloides*, *S. baltica*, *V. anguillarum*, and *V. parahaemolyticus.* Its active agents also prevented infections of both fish-associated pathogens and food spoiling bacteria, which means it can not only help in the disease control mechanism but also in improving the safety of food by reduction of the microbial contamination.

## 1. Introduction

Bacterial fish diseases are caused by an ample number of bacterial pathogens which negatively affect fish health and productivity and also cause high economic loss among cultured freshwater and marine fishes [1,2]. These diseases harmfully affect the mariculture industry [3], especially in the Mediterranean regions such as Türkiye. Bacterial pathogens also affect the freshwater fauna [4]. A wide range of gram-positive (G + ve) and gram-negative (G − ve) bacteria are involved in causing devastating fish diseases [5]. In Türkiye, numerous G + ve and G − ve bacterial pathogens have been isolated, identified, and characterized from diseased fish such as *Aeromonas sobria* [6], *Yersinia ruckeri* [7], *Lactococcus garvieae* [8], *Mycobacterium marinum* [9], *Vagococcus salmoninarum* [10], *Vibrio gigantis* strain C24 [11], *Plesiomonas shigelloides* [12], staphylococcal infections [13], and *Pseudomonas* species [14], among several others. From another side, there are abundant food poisoning bacterial strains that contaminate fish and shellfish and their products or are naturally present in the flora of fish and shellfish and pose significant health risks to human beings and leads to foodborne diseases and serious infection outbreaks [15,16].

In aquaculture, the commonly used method to control bacterial diseases is the use of antibiotics and antimicrobial agents [17]. However, their indiscriminate overuse may lead to serious problems such as (a) the development of antimicrobial resistance which demonstrates a crisis concern [18,19], (b) negative environmental impacts [20,21], and (c) the presence of drug residues in fish fillets which can produce increased human risks [22,23,24]. Because of the above-mentioned problems, there is an urgent need to find antibiotic alternatives to control bacterial diseases [25]. Plant-based applications (e.g., plants, herbal extracts, essential oils, and phytochemicals) are considered as important and effective control measures for bacterial fish diseases because of their immune-stimulating and antimicrobial efficacy [26,27].

Investigation of the antimicrobial efficacy of plants, extracts, herbal medicines, or essential oils extracted from plants has flourished in the past years because of their effectual results against a range of bacterial pathogens [28,29]. Dawood and coauthors have reviewed the most commonly used herbal essential oils (EOs) which possess unique antibacterial and antiparasitic effects, and they elucidated their positive roles for use in maintaining aquaculture sustainability [30]. Several reports also defined the antifungal efficacy of some herbal plants [31], specifically against the yeast strain *Candida albicans* [32]. Furthermore, several herbal supplements can be efficiently used to combat diseases in aquaculture [33]. Researchers and scientists have found that the antimicrobial activities of the herbal supplements are closely related to (and/or attributed to) their phytochemical contents which possess potent antimicrobial effects [34].

Olive oil production is an essential agricultural economic resource for a vast number of Mediterranean countries, including Türkiye [35]. However, the production processes generate significant amounts of olive oil wastewater (OMWW) [36]. OMWW is known as a significant source of environmental hazards because of its high organic load and phenolic substance content [37,38]. Recent studies demonstrated that the valuable phenolic compounds of OMWW can be used in various industrial and food-based applications [39,40,41,42]. Of interest, it was also found that the polyphenols extracted from OMWW can effectively and successfully be used for the treatment of human dermal disorders [43], besides the recovery of natural antioxidant supplements [44] and beneficial nutraceuticals [45]. It was demonstrated that the solvent extraction method has been used to effectively collect the valuable components from OMWW [46]. Research studies elucidated that solvent extraction methods can effectively increase the antioxidant and antimicrobial activities of OMWW [39,47,48,49].

One of the most important effects of OMWW is its antimicrobial characteristics. Research studies demonstrated that OMWW produced strong antimicrobial effects against several bacterial isolates and *C. albicans* [50,51]. Moreover, the OMWW extract also induced potent inhibitory effects against *Pseudomonas fluorescens* obtained from Mozzarella cheese [52]. In aquaculture, the use of OMWW extracts may effectively combat bacterial pathogens. OMWW may also be used as a true-to-life antimicrobial drug in sustainable aquaculture and processing technology applications. Previous studies have shown that OWMM improves the nutritional quality of fish meat fillets [53] and supports the antioxidant defensive mechanisms of fish [54,55], their growth performance, and their immune responses [56]. However, studies have yet to be found in the literature on using OWMM for antimicrobial activities against fish-associated pathogens or foodborne pathogenic bacteria. Therefore, this study was designed to explore the antimicrobial activities of an ethanolic extract prepared from OMWW against 38 bacterial strains and a yeast strain, *C. albicans*. The results of this study will be leading and will help to maintain aquaculture sustainability and provide a safe and antibiotic-free aquatic environment.

## 2. Materials and Methods

### 2.1. Olive Mill Wastewater (OMWW) Extract

The OMWW extract was obtained as a by-product from a local factory situated in Çanakkale, Türkiye. The OMWW was transported in 20 L plastic containers, delivered to the laboratory within approximately one hour, and immediately subjected to lyophilization. Subsequently, 10 g of the dried OMWW powder were dissolved in 100 mL of 96% ethanol for one hour and then left at room temperature [57]. The mixture was centrifuged with a high-speed centrifuge at 16,000× *g* for 6 min [58]. After that, the mixture was then filtered with sterile syringe filters (0.45 μm), the sediment was discarded, and the supernatant was collected and then refrigerated and stored at −20 °C for further in vitro antimicrobial analysis.

### 2.2. GC-MS Analysis

The ingredients of OMWW extract were examined using Gas chromatography/mass spectrometry (GC-MS) analysis (Shimadzu GCMS QP 2010 ULTRA, Kyoto, Japan). The separation of the components present in this extract was carried out using a Rxi-5 ms capillary column (30 m; 0.25 mm; 0.25 µm) (Restek Corporation, Bellefonte, PA, USA) with a low polarity phase diphenyl dimethyl polysiloxane and helium as the carrier gas. The oven temperature was initially set to 40 °C. After holding at this temperature for 3 min, it was increased at a rate of 4 °C per minute up to 240 °C. The injection was carried out in split mode (ratio 25) at 250 °C with an injection volume of 1 µL. The mass spectrum (70 eV) was set to scan in the m/z range of 40–450 amu, with a column flow rate of 1.78 mL/min, a pressure of 100 kPa, and a total flow rate of 49.3 mL/min. The GC-MS analysis was completed in a total of 53 min. The chromatograms of the tested OMWW extract were compared with the Wiley W9N11 mass spectral library to identify their components.

### 2.3. Bacterial and Yeast Strains Used in This Study

This study investigated the antimicrobial effects of OMWW extract against 38 bacterial strains and one yeast strain (*C. albicans*). A total of 27 bacterial strains used in the current study have been obtained from the bacterial culture collection unit that is located at Çanakkale Onsekiz Mart University, Faculty of Marine Sciences and Technology, Türkiye (as shown in Table 1). The bacterial isolates, fish source, strain ID, their NCBI GenBank accession numbers, and culture characteristics have been previously published in our paper by Yilmaz et al. [59]. In addition, the bacterial isolate, *V. gigantis* C24 and its accession numbers has been retrieved also from our recently published paper by Yilmaz et al. [11]. A total of 10 reference bacterial isolates and one yeast isolate (*C. albicans*) of the collection type and their ID have been also used as listed in Table 2. The culture media and culture characteristics have been previously published in detail by Yilmaz et al. [59]. The bacterial and yeast strains were then preserved and stored at −80 °C in cryo tubes with culture media and 20% glycerol for long-term preservation until being used for the antimicrobial analysis tests.

### 2.4. In Vitro Antimicrobial Analysis

The disk diffusion test, minimum inhibitory concentration (MIC), and minimum bactericidal/fungicidal concentration (MBC/MFC) analyses were used to evaluate the antimicrobial efficacy of OMWW extract.

#### 2.4.1. Disk Diffusion Test

The antimicrobial effects of the OMWW extract on different bacterial isolates were determined using Kirby-Bauer’s disc diffusion method. The bacterial isolates were cultured in appropriate liquid media at different culture temperatures as specified before by Yilmaz et al. [59]. The bacterial densities were adjusted to 0.5 McFarland standards and transferred using sterile cotton swabs onto suitable solid culture media. Disks loaded with 2 mg of OMWW extract in 20 μL were dried under aseptic conditions and placed onto inoculated solid media. The inoculations were performed in triplicate (n = 3). The plates were then incubated for 24 h. After that, post-incubation inhibition zones were measured, with diameters less than 12 mm considered ineffective, between 12 and 20 mm as moderate, and over 20 mm as highly effective according to the guidelines of the methods described in [60].

#### 2.4.2. MIC and MBC/MFC Analysis

The MIC analysis was conducted according to the method specified by the Clinical and Laboratory Standards Institute [61]. Briefly, a 4000 μg/mL stock solution of OMWW extract was prepared in a suitable growth medium containing 5% DMSO. Subsequently, 50 µL of the growth medium was added to each well of a 96-well plate, followed by the addition of 50 µL of the OMWW extract stock solution. Two-fold serial dilutions were then performed across the plate, resulting in final concentrations of the OMWW extract ranging from 4000 to 0.977 μg/mL. To each well, 50 µL of a bacterial suspension (10⁵ CFU/mL) was added. The control wells included the following: 100 µL of growth medium with bacteria, 100 µL of growth medium containing DMSO (5%), OMWW extract without bacteria, and 100 µL of growth medium without bacteria. The plates were then incubated at an appropriate growth temperature for 24 h. The MIC values were determined by observing the lowest OMWW extract concentration inhibiting the visible bacterial growth. Samples were taken from the MIC wells, and the two preceding dilutions were plated onto solid media to determine the MBC/MFC. The MBC/MFC was assigned as the lowest concentration of OMWW extract that kills ≥ 99.9% of the initial bacterial and Candida inoculum and results in no growth on the agar plate.

## 3. Results

### 3.1. GC-MS Analysis of the Constituents in OMWW Extract

Based on the GC-MS analysis, the main constituents of the OMWW extract with percentages above 5% were oleic acid (24.82%), 9-Borabicyclo[3.3.1]nonane 9-ethoxy- (11.82%), palmitic acid (8.61%), Oxazole, 5-methyl-2-phenyl- (8.03%)), Beta-pinenoxid (6.25%), and Octopamine (6.13%) (as shown in Table 3).

### 3.2. Disc Diffusion, MIC, and MBC/MFC Results

This study tested the OMWW extract in vitro on 38 different bacteria and one yeast strain, *C. albicans*. The in vitro antimicrobial effect results on fish-associated pathogens and non-pathogenic isolates are depicted in Table 4 and Table 5. The disc diffusion test results showed that the OMWW extract was effective against one gram-positive (Table 4) and eight gram-negative (Table 5) bacterial species. The highest antimicrobial inhibitory zone diameter of 20 mm was observed against *S. baltica* strain SY-S145. In comparison, the lowest inhibition zone diameter of 8 mm was noted against *A. sobria* SY-AS3, *Lysinibacillus xylanilyticus* SY-LX12, *A. sobria* SY-AS1, and *A. salmonicida* ATCC 33658. Of interest, the OMWW extract inhibition zone was observed with a diameter of 16 mm against *P. shigelloides* SY-PS16, 14 mm against *V. anguillarum* SY-L24, and 12 mm against *V. gigantis*.

In this study, the OMWW extract demonstrated significant antimicrobial activities against several bacterial strains with MIC and MBC values of 1000 µg/mL and below. Specifically, the OMWW extract was effective against *S. baltica* SY-S145, with MIC and MBC values at 500 µg/mL. Similarly, this extract inhibited *V. anguillarum* SY-L24 and *V. gigantis* C24 with MIC and MBC values of 500 µg/mL. Furthermore, it demonstrated an MIC at 1000 µg/mL and MBC at 2000 µg/mL against *A. sobria* SY-AS3. In the case of *P. shigelloides* SY-PS16, the MIC was at 1000 µg/mL and MBC was at 2000 µg/mL. Interestingly, its results for *A. salmonicida* ATCC 33658 were an MIC at 500 µg/mL and MBC at 1000 µg/mL, and for *V. parahaemolyticus* ATCC 17802, they were an MIC and MBC at 1000 µg/mL.

## 4. Discussion

Globally, several countries banned the indiscriminate use and application of antibiotics in aquaculture [62]. It was found that heavy use of antibiotics will lead to serious problems for human and animal health [63]. Development of antibiotic-resistant bacteria, drug residues in fish flesh, environmental contamination, and high costs are among the causes that render the use of antibiotics prohibited [64]. Therefore, researchers and scientists conducted several investigations and experimental trials seeking antibiotic alternatives. Herbal medicines, plant extracts, and phytochemicals are on the top of the list for these alternatives because of their durability, safety, and efficacy [65,66]. They are considered as an effective way to control diseases in aquaculture [27,67] and enhance the immune responses against the challenging pathogens [26,68].

In the present study, based on the GC-MS analysis, it was found that the main phytochemical constituents of the OMWW extract are oleic acid (24.82%) and palmitic acid (8.61%), both of which have been found to have antibacterial and antifungal effects, as reported by previously published studies [69,70]. However, for the other components identified, such as 9-Borabicyclo[3.3.1]nonane 9-ethoxy- (11.82%), Oxazole, 5-methyl-2-phenyl- (8.03%), Beta-pinenoxid (6.25%), and Octopamine (6.13%), our literature review did not find any published study about antimicrobial or antifungal activities, which need future research and investigations.

The present study results showed that the OMWW extract exhibited a strong antimicrobial effects against the opportunistic human pathogen *S. baltica* [71], which is known to be responsible for spoilage in aquaculture products and can lead to food poisoning from fish and shrimp [16,71]. In addition, the OMWW extract demonstrated an antimicrobial effect on fish-associated bacterial pathogens such as *P. shigelloides*, *V. anguillarum*, and *V. gigantis*, all of which are associated with significant economic losses and fish mortalities in aquaculture operations [11,72,73]. *P. shigelloides* and *V. anguillarum* have been reported to cause gastrointestinal infections [74,75] and sepsis in humans [73]. Roila et al. [76] reported that OMWW polyphenol extract was effective against an ample number of bacterial pathogens such as *Pseudomonas aeruginosa*, *Brochothrix thermosphacta*, *Lactobacillus sakei* subsp. *sakei*, *Shewanella putrefaciens*, *E. coli*, *Lactococcus lactis*, *Pseudomonas fluorescens*, and *Lactobacillus plantarum*. This study showed that MIC values ranged from 15.6 to 125 mg/mL, and MBC values ranged from 15.6 to 250 mg/mL [76]. Roila et al. [52] reported that an OMWW extract showed antimicrobial effects against 64 *Pseudomonas fluorescens* strains, with MIC50 and MIC90 values of 5 mg/mL and 7 mg/mL, and MBC50 and MBC90 values of 6 mg/mL and 8 mg/mL, respectively. In a study performed on clinical isolates of *Pseudomonas aeruginosa*, *Klebsiella pneumoniae*, *Acinetobacter baumannii*, *Morganella morganii*, *Proteus stuartii*, *Serratia marcescens*, *Stenotrophomonas maltophilia*, *E. coli*, and *Pseudomonas syringae*, the MIC values of olive mill wastewater ranged between 8 and 16 mg/mL [77]. The published literature showed that the MIC or MBC values of OMWW extracts obtained without solvents are higher than those obtained in this study. Similarly, in our findings, MIC values of 0.15–0.3 mg/mL and MBC values of 0.3 mg/mL were reported against *Campylobacter* species using a spray-dried OMWW extract [78].

Aeromonas species have been reported to cause serious infections in humans and animals [79]. It has been reported many times that *Aeromonas* species cause economic losses in carp [80], Nile tilapia [81], trout, and many other fish species [72]. In this study, the antimicrobial effects of an OMWW extract on *A. sobria* SY-AS1 and *A. salmonicida* ATCC 33658 showed ineffective inhibition with an 8 mm zone. However, interestingly, the MIC for *A. salmonicida* subsp. *salmonicida* ATCC 33658 was determined to be 500 µg/mL, demonstrating that disk diffusion test results do not always correlate with MIC or MBC results. Similarly, Yılmaz et al. [59] found a contrast between the disk diffusion test result and MIC values for the *Acinetobacter johnsonii* SY-AJ isolate, where the inhibition zone was 8 mm, but the MIC value was 500 μg/mL, showing a difference between the two methods.

Previous research studies demonstrated that the antimicrobial efficacy of olive mill wastewaters may be linked to the presence of high amount of polyphenols and phenolic compounds [82,83]. Abu-Lafi et al. [51] reported that the phenolic compounds present in OMWW, such as hydroxytyrosol, oleuropein, and tyrosol, presented high antimicrobial and antioxidant effects. Moreover, Yakhlef et al. [83] found that the presence of glutaraldehyde-like compounds in olive products exerted powerful antimicrobial activities. Hydroxytyrosol and oleuropein that are present in olive products were found to possess cytotoxic activities against ATCC reference bacterial strains [84]. The proposed antimicrobial mechanism action of the polyphenols and phenolic compounds present in olive products targets the bacterial cell membranes [84] or other bacterial components [85]. Accordingly, OMWW could be considered as a valuable source of natural antimicrobial compounds. However, the precise antimicrobial effects of phytochemicals present in olive products require additional research studies.

## 5. Conclusions and Future Research

To put it briefly, while the OMWWE showed moderate inhibitory effects against significant fish pathogenic bacteria such as *P. shigelloides* and *V. anguillarum*, it exhibited solid antimicrobial effects against *Shewanella baltica*, which can cause food spoilage. The absence of antimicrobial effects on natural flora and probiotic bacteria is a significant result. Our findings suggest that future studies could integrate probiotic bacteria with OMWWE in feed and conduct in vivo studies. In addition, challenge tests in which OMWWE is applied to inoculated sterile meat should also be conducted to evaluate the practical efficacy of OMWWE against foodborne bacterial pathogens, providing further evidence of its potential as a natural antimicrobial agent for food preservation. Moreover, some researchers have considered antimicrobial activity present when MIC values are 1000 µg/mL or below [86]. Thus, although the disk diffusion results in this study did not indicate antimicrobial activity, we cannot ignore the MIC values of 1000 µg/mL that we determined for *A. veronii* SY-AV10, *A. hydrophila* SY-AH2, and *V. parahaemolyticus* ATCC 17802. Therefore, further research is also necessitated to explore the addition of OMWWE to fish and shellfish seafood products to prevent and control several bacterial pathogens such as *A. veronii*, *A. hydrophila*, *P. shigelloides*, *S. baltica*, *V. anguillarum*, and *V. parahaemolyticus*.

## Figures and Tables

**Table 1:** Fish-associated bacterial pathogens that were provided from our laboratory, their ID, and their NCBI GenBank accession numbers.

Bacterial Isolates	Strain ID	Fish Source	Accession Numbers	Previously Published in
*Aeromonas sobria* (*A. sobria*)	SY-AS3	*Onchorynchus mykiss*	KY126831.1	[59]
*A. sobria*	SY-AS1	*Cyprinus carpio*	KY126835.1	[59]
*A. veronii*	SY-AV10	*Oreochromis niloticus*	MG563680.1	[59]
*A. hydrophila*	SY-AH2	*O. niloticus*	MG844996.1	[59]
*Acinetobacter johnsonii*	SY-AJ	*O. mykiss*	KY126836.1	[59]
*Niallia nealsonii*	SY-BN9	*O. mykiss*	KY126832.1	[59]
*Bacillus pumilus (B. pumilus)*	SY-BP15	*O. mykiss*	KY126833.1	[59]
*B. subtilis*	SY-BS19	*O. mykiss*	KY077683.1	[59]
*B. subtilis*	SY-BS20	*O. mykiss*	KY078783.1	[59]
*B. thuringiensis*	SY-BT11	*O. mykiss*	KY127450.1	[59]
*Citrobacter freundii*	SY-CF14	*O. mykiss*	KY126840.1	[59]
*Enterobacter* sp.	SY-EN560	*O. mykiss*	KX388232.1	[59]
*Lelliottia amnigena*	SY-LA18	*O. mykiss*	KY126834.1	[59]
*Lysinibacillus xylanilyticus*	SY-LX12	*O. mykiss*	KY078822.1	[59]
*Pseudomonas putida*	SY-PP21	*O. mykiss*	KY126830.1	[59]
*Shewanella baltica*	SY-S145	*O. mykiss*	KX388237.1	[59]
*Flavobacterium tructae*	SY-FS1	*O. mykiss*	KY386294.1	[59]
*E. coli*	SY-EC4	*Symphysodon* spp.	MG855664.1	[59]
*Klebsiella pneumoniae*	SY-KP1	*Symphysodon* spp.	MG855663.1	[59]
*Citrobacter* sp.	SY-C10	*O. niloticus*	KX388233.1	[59]
*Edwardsiella tarda (E. tarda)*	SY-ED1	*O. niloticus*	KY126838.1	[59]
*E. tarda*	SY-ED14	*O. niloticus*	KX388234.1	[59]
*Lactococcus garvieae*	SY-LG1	*O. mykiss*	KY118086.1	[59]
*Plesiomonas shigelloides (P. shigelloides)*	SY-PS16	*O. mossambicus × O. niloticus*	MG574356.1	[59]
*Vibrio anguillarum*	SY-L24	*Dicentrarchus labrax*	KX388236.1	[59]
*V. gigantis*	C24	*D. labrax*	ON778781.1	[11]
			ON792326.1	
*Yersinia ruckeri*	E42	*O. mykiss*	KX388238.1	[59]

**Table 2:** Reference bacterial and yeast strains (collection strain) that were used in the present study.

Bacterial and Yeast Isolates	Strain ID	Source
*A. salmonicida* subsp. *salmonicida*	ATCC 33658	Collection strain
*V. parahaemolyticus*	ATCC 17802	Collection strain
*Klebsiella pneumoniae*	ATCC BAA-1144	Collection strain
*Staphylococcus aureus*	ATCC 25923	Collection strain
*Salmonella enterica* subsp. *enterica* serovar *enteritidis*	ATCC 13076	Collection strain
*Pseudomonas aeruginosa*	ATCC 10145	Collection strain
*Streptococcus iniae*	ATCC 29177	Collection strain
*Lactobacillus plantarum*	BC 7321	Collection strain
*B. spizizenii*	ATCC 6633	Collection strain
*E. coli*	ATCC 25922	Collection strain
*Candida albicans* (*C. albicans*)	ATCC 10231	Collection strain

**Table 3:** The main constituents of the OMWW extract that were identified via GC-MS analysis.

Peak	Retention Time	Area %	Ingredients
1.	13.38	4.22	2 Ethyl hexanol
2.	17.76	0.97	2,3-Dihydro-3,5-dihydroxy-6-methyl-4H-pyran-4-one
3.	20.73	0.85	2,3-Dihydro-benzofuran
4.	25.9	0.2	Benzoic acid, 4-formyl-, methyl ester
5.	27.51	1.18	4-Methoxy-2,5-dihydrotoluene
6.	27.69	1.12	4,6-Heptadienoic acid, 3,3,6-trimethyl-, ethyl ester
7.	28.1	1.11	Benzeneethanol, 4-hydroxy-
8.	28.39	0.37	9-Oxabicyclo[6.1.0]nonane, 1-methyl-, cis-
9.	28.6	0.54	2-Hydroxy-4-methylbenzaldehyde
10.	30.96	0.51	4-Methyl-5-penta-1,3-dienyltetrahydrofuran-2-one
11.	31.18	6.25	Beta-pinenoxid
12.	32.07	0.52	2-Butyl-5-methyl-3-(2-methylprop-2-enyl)cyclohexanone
13.	33.3	0.76	4-Hydroxy-4-(2-methylcyclohexyl)butan-2-one
14.	35.51	6.13	Octopamine
15.	35.6	3.31	Benzeneacetic acid, 3,4-dihydroxy-
16.	35.89	1.33	2-Oxabicyclo[4.3.0]nonan-3-one, 8,9-dihydroxy-4-methyl-, (Z)
17.	36.14	0.88	1-Cyclooctene-1-carboxylic acid
18.	36.43	3.56	1,5-Hexadiene-3,4-diol, 2,5-dimethyl-
19.	37.03	11.82	9-Borabicyclo[3.3.1]nonane, 9-ethoxy-
20.	37.2	0.46	3-(3-Butenyl)-2-cycloocten-1-one
21.	37.69	8.03	Oxazole, 5-methyl-2-phenyl- (
22.	38.11	1.25	Tetradecanoic acid
23.	38.23	0.74	4-Methylphenylthioacetaldehyde diethyl acetal
24.	38.6	0.24	5,7-Octadien-3-ol, 2,4,4,7-tetramethyl-, (E)-
25.	38.77	0.34	cis-3a,4,5,6,7,7a-hexahydro-5,5-dimethyl-1H-inden-1-one
26.	38.98	1.57	2(3H)-Naphthalenone,4,4a,5,6,7,8-hexahydro-4a-methyl-
27.	40.65	0.35	2,7-Dioxatricyclo[4.4.0.0(3,8)]decan-4-amine, stereoisomer
28.	42.08	0.45	Lidocaine
29.	42.53	0.93	Tricyclo[8.6.0.0(2,9)]hexadeca-8,16,head,tail-dione, trans-2,9-cisoid-9,10-cis-1,10
30.	42.86	0.58	cis-9-Hexadecenoic acid
31.	43.44	8.61	Palmitic acid
32.	43.6	0.54	Naphth[2,3-b]oxirene, decahydro-
33.	44.19	0.95	Hexadecanoic acid, ethyl ester
34.	47.79	24.82	Oleic Acid
35.	48.34	3.44	Ethyl Oleate
36.	52.62	1.05	9-Octadecenamide

**Table 4:** Disc diffusion, MIC, and MBC results of OMWW extract against gram-positive bacteria.

Gram-Positive Bacteria	OMWW Extract		
	2 mg/disc	MIC (µg/mL)	MBC (µg/mL)
*Niallia nealsonii* SY-BN9	-	>2000	>2000
*B. pumilus* SY-BP15	-	>2000	>2000
*B. subtilis* SY-BS19	-	>2000	>2000
*B. subtilis* SY-BS20	-	>2000	>2000
*B. thuringiensis* SY-BT11	-	>2000	>2000
*B. spizizenii* ATCC 6633	-	>2000	>2000
*Lysinibacillus xylanilyticus* SY-LX12	8	>2000	>2000
*Lactococcus garvieae* SY-LG1	-	>2000	>2000
*Lactobacillus plantarum* BC 7321	-	>2000	>2000
*Streptococcus iniae* ATCC 29177	-	>2000	>2000
*Staphylococcus aureus* ATCC 25923	-	2000	>2000

**Table 5:** Disc diffusion, MIC, and MBC/MFC* results of OMWW extract against gram-negative bacteria and *C. albicans* *.

Organisms	OMWW Extract		
	2 mg/disc	MIC (µg/mL)	MBC (µg/mL)
*A. sobria* SY-AS3	8	1000	2000
*Acinetobacter johnsonii* SY-AJ	-	>2000	>2000
*Citrobacter freundii* SY-CF14	-	>2000	>2000
*Enterobacter* sp. SY-EN560	-	>2000	>2000
*Lelliottia amnigena* SY-LA18	-	>2000	>2000
*Pseudomonas putida* SY-PP21	-	>2000	>2000
*Shewanella baltica* SY-S145	20	500	500
*Flavobacterium tructae* SY-FS1	-	>2000	>2000
*E. coli* SY-EC4	-	>2000	>2000
*Klebsiella pneumoniae* SY-KP1	-	>2000	>2000
*A. sobria* SY-AS1	8	2000	>2000
*A. veronii* SY-AV10	-	1000	>2000
*A. hydrophila* SY-AH2	-	1000	>2000
*Citrobacter* sp. SY-C10	-	>2000	>2000
*E. tarda* SY-ED1	-	>2000	>2000
*E. tarda* SY-ED14	-	>2000	>2000
*P. shigelloides* SY-PS16	16	1000	2000
*V. anguillarum* SY-L24	14	500	500
*V. gigantis* C24	12	500	500
*Yersinia ruckeri* E42	-	2000	>2000
*A. salmonicida* ATCC 33658	8	500	1000
*V. parahaemolyticus* ATCC 17802	-	1000	1000
*Klebsiella pneumoniae* ATCC BAA-1144	-	>2000	>2000
*Salmonella enterica* subsp. *enterica serovar* enteritidis ATCC 13076	-	>2000	>2000
*Pseudomonas aeruginosa* ATCC 10145	-	>2000	>2000
*E. coli* ATCC 25922	-	>2000	>2000
*C. albicans* ATCC 10231	-	>2000 *	>2000 *

* means yeast strain.

## Data Availability

The original contributions presented in the study are included in the article, further inquiries can be directed to the corresponding authors.

## References

[1] Abdel-Latif H.M.R., Citarasu T., Turgay E., Yilmaz E., Yousefi M., Shekarabi P.H., Ahmadifar E., Nowosad J., Kucharczyk D., Yilmaz S. (2024). Control of yersiniosis in rainbow trout, : Innovative non-antibiotic feed-based strategies. Ann. Anim. Sci..

[2] El-Son M.A.M., Nofal M.I., Abdel-Latif H.M.R. (2021). Co-infection of *Aeromonas hydrophila* and *Vibrio parahaemolyticus* isolated from diseased farmed striped mullet (*Mugil cephalus*) in Manzala, Egypt—A case report. Aquaculture.

[3] Toranzo A.E., Magariños B., Romalde J.L. (2005). A review of the main bacterial fish diseases in mariculture systems. Aquaculture.

[4] Zaheen Z., War A.F., Ali S., Yatoo A.M., Ali M.N., Ahmad S.B., Rehman M.U., Paray B.A., Dar G.H., Bhat R.A., Qadri H., Al-Ghamdy K.M., Hakeem K.R. (2022). Chapter 7—Common bacterial infections affecting freshwater fish fauna and impact of pollution and water quality characteristics on bacterial pathogenicity. Bacterial Fish Diseases.

[5] Varalakshmi B., Shanmugapriya A., Karpagam T., Suganya V., Firdous J., Arumugam V.A., Sridevi R., Abinaya M., Saradhasri V., Balasubramanian B., Liu W.-C., Sattanathan G. (2022). Bacterial Fish Diseases and Treatment. Aquaculture Science and Engineering.

[6] Kubilay A., Didinen B.I., Metin S., Onuk E.E., Koca S.B., Ulukoy G. (2020). First record of *Aeromonas sobria* in yellow tail cichlid, *Pseudotropheus acei*. Bull. Eur. Assoc. Fish Pathol..

[7] Onuk E.E., Didinen B.I., Giftci A., Yardimci B., Pekmezci G.Z. (2019). Molecular characterisation of antibiotic resistance in Yersinia ruckeri isolates from Turkey. Bull. Eur. Assoc. Fish Pathol..

[8] Didinen B.I., Yardimci B., Onuk E.E., Metin S., Yildirim P. (2014). Naturally *Lactococcus garvieae* infection in rainbow trout (*Oncorhyncus mykiss* Walbaum, 1792): New histopathological observations, phenotypic and molecular identification. Rev. De Med. Vet..

[9] Avsever M.L., Çavuçoʇlu C., Günen M.Z., Yazicioʇlu Ö., Eskiizmirliler S., Didinen B.I., Tunaligil S., Erdal G., Özden M. (2014). The first report of *Mycobacterium marinum* isolated from cultured meagre, *Argyrosomus regius*. Bull. Eur. Assoc. Fish Pathol..

[10] Didinen B.I., Kubilay A., Diler O., Ekici S., Onuk E.E., Findik A. (2011). First isolation of *Vagococcus salmoninarum* from cultured rainbow trout (*Oncorhynchus mykiss*, Walbaum) broodstocks in Turkey. Bull. Eur. Assoc. Fish Pathol..

[11] Yilmaz S., Karataş S., Steinum T.M., Gürkan M., Yilmaz D.K., Abdel-Latif H.M.R. (2023). Isolation, Identification, and Pathogenicity of *Vibrio gigantis* Retrieved from European Seabass (*Dicentrarchus labrax*) Farmed in Türkiye. Animals.

[12] Duman M., García Valdés E., Ay H., Altun S., Saticioglu I.B. (2023). Description of a Novel Fish Pathogen, Plesiomonas shigelloides subsp. oncorhynchi, Isolated from Rainbow Trout (*Oncorhynchus mykiss*): First Genome Analysis and Comparative Genomics. Fishes.

[13] Çanak Ö., Timur G. (2020). An initial survey on the occurrence of staphylococcal infections in Turkish marine aquaculture (2013–1014). J. Appl. Ichthyol..

[14] Duman M., Mulet M., Altun S., Saticioglu I.B., Ozdemir B., Ajmi N., Lalucat J., García-Valdés E. (2021). The diversity of Pseudomonas species isolated from fish farms in Turkey. Aquaculture.

[15] Ghaly A.E., Dave D., Budge S., Brooks M. (2010). Fish spoilage mechanisms and preservation techniques. Am. J. Appl. Sci..

[16] Gram L., Dalgaard P. (2002). Fish spoilage bacteria—Problems and solutions. Curr. Opin. Biotechnol..

[17] Snieszko S.F. (1954). Therapy of Bacterial Fish Diseases. Trans. Am. Fish. Soc..

[18] Santos L., Ramos F. (2018). Antimicrobial resistance in aquaculture: Current knowledge and alternatives to tackle the problem. Int. J. Antimicrob. Agents.

[19] Preena P.G., Swaminathan T.R., Kumar V.J.R., Singh I.S.B. (2020). Antimicrobial resistance in aquaculture: A crisis for concern. Biologia.

[20] Cao L., Wang W., Yang Y., Yang C., Yuan Z., Xiong S., Diana J. (2007). Environmental impact of aquaculture and countermeasures to aquaculture pollution in China. Environ. Sci. Pollut. Res. Int..

[21] Ahmad A.L., Chin J.Y., Mohd Harun M.H.Z., Low S.C. (2022). Environmental impacts and imperative technologies towards sustainable treatment of aquaculture wastewater: A review. J. Water Process Eng..

[22] Liu X., Steele J.C., Meng X.-Z. (2017). Usage, residue, and human health risk of antibiotics in Chinese aquaculture: A review. Environ. Pollut..

[23] He Z., Cheng X., Kyzas G.Z., Fu J. (2016). Pharmaceuticals pollution of aquaculture and its management in China. J. Mol. Liq..

[24] He X., Deng M., Wang Q., Yang Y., Yang Y., Nie X. (2016). Residues and health risk assessment of quinolones and sulfonamides in cultured fish from Pearl River Delta, China. Aquaculture.

[25] Newaj-Fyzul A., Austin B. (2015). Probiotics, immunostimulants, plant products and oral vaccines, and their role as feed supplements in the control of bacterial fish diseases. J. Fish Dis..

[26] Elumalai P., Kurian A., Lakshmi S., Faggio C., Esteban M.A., Ringø E. (2020). Herbal Immunomodulators in Aquaculture. Rev. Fish. Sci. Aquac..

[27] Reverter M., Bontemps N., Lecchini D., Banaigs B., Sasal P. (2014). Use of plant extracts in fish aquaculture as an alternative to chemotherapy: Current status and future perspectives. Aquaculture.

[28] Munuswamy H., Thirunavukkarasu T., Rajamani S., Elumalai E.K., Ernest D. (2013). A review on antimicrobial efficacy of some traditional medicinal plants in Tamilnadu. J. Acute Dis..

[29] Mahasneh A.M., El-Oqlah A.A. (1999). Antimicrobial activity of extracts of herbal plants used in the traditional medicine of Jordan. J. Ethnopharmacol..

[30] Dawood M.A.O., El Basuini M.F., Zaineldin A.I., Yilmaz S., Hasan M.T., Ahmadifar E., El Asely A.M., Abdel-Latif H.M.R., Alagawany M., Abu-Elala N.M. (2021). Antiparasitic and Antibacterial Functionality of Essential Oils: An Alternative Approach for Sustainable Aquaculture. Pathogens.

[31] Samadi F.M., Suhail S., Sonam M., Sharma N., Singh S., Gupta S., Dobhal A., Pradhan H. (2019). Antifungal efficacy of herbs. J. Oral Biol. Craniofacial Res..

[32] Hsu H., Sheth C.C., Veses V. (2021). Herbal extracts with antifungal activity against Candida albicans: A systematic review. Mini Rev. Med. Chem..

[33] Li M., Wei D., Huang S., Huang L., Xu F., Yu Q., Liu M., Li P. (2022). Medicinal herbs and phytochemicals to combat pathogens in aquaculture. Aquac. Int..

[34] Aqil F., Zahin M., Ahmad I., Owais M., Khan M.S.A., Bansal S.S., Farooq S., Ahmad I., Owais M., Shahid M., Aqil F. (2010). Antifungal Activity of Medicinal Plant Extracts and Phytocompounds: A Review. Combating Fungal Infections: Problems and Remedy.

[35] Ruiz-Carrasco B., Fernández-Lobato L., López-Sánchez Y., Vera D. (2023). Life Cycle Assessment of Olive Oil Production in Turkey, a Territory with an Intensive Production Project. Agriculture.

[36] Dermeche S., Nadour M., Larroche C., Moulti-Mati F., Michaud P. (2013). Olive mill wastes: Biochemical characterizations and valorization strategies. Process Biochem..

[37] Khdair A., Abu-Rumman G. (2020). Sustainable Environmental Management and Valorization Options for Olive Mill Byproducts in the Middle East and North Africa (MENA) Region. Processes.

[38] Roig A., Cayuela M.L., Sánchez-Monedero M.A. (2006). An overview on olive mill wastes and their valorisation methods. Waste Manag..

[39] Foti P., Romeo F.V., Russo N., Pino A., Vaccalluzzo A., Caggia C., Randazzo C.L. (2021). Olive Mill Wastewater as Renewable Raw Materials to Generate High Added-Value Ingredients for Agro-Food Industries. Appl. Sci..

[40] Rocha C., Soria M.A., Madeira L.M. (2022). Olive Mill Wastewater Valorization through Steam Reforming Using Multifunctional Reactors: Challenges of the Process Intensification. Energies.

[41] Ahmadifar E., Yousefi M., Karimi M., Fadaei Raieni R., Dadar M., Yilmaz S., Dawood M.A.O., Abdel-Latif H.M.R. (2021). Benefits of Dietary Polyphenols and Polyphenol-Rich Additives to Aquatic Animal Health: An Overview. Rev. Fish. Sci. Aquac..

[42] Naiel M.A.E., El-Kholy A.I., Negm S.S., Ghazanfar S., Shukry M., Zhang Z., Ahmadifar E., Abdel-Latif H.M.R. (2023). A Mini-Review on Plant-Derived Phenolic Compounds with Particular Emphasis on Their Possible Applications and Beneficial Uses in Aquaculture. Ann. Anim. Sci..

[43] Carrara M., Kelly M.T., Roso F., Larroque M., Margout D. (2021). Potential of Olive Oil Mill Wastewater as a Source of Polyphenols for the Treatment of Skin Disorders: A Review. J. Agric. Food Chem..

[44] Agalias A., Magiatis P., Skaltsounis A.-L., Mikros E., Tsarbopoulos A., Gikas E., Spanos I., Manios T. (2007). A New Process for the Management of Olive Oil Mill Waste Water and Recovery of Natural Antioxidants. J. Agric. Food Chem..

[45] Cuffaro D., Bertolini A., Bertini S., Ricci C., Cascone M.G., Danti S., Saba A., Macchia M., Digiacomo M. (2023). Olive Mill Wastewater as Source of Polyphenols with Nutraceutical Properties. Nutrients.

[46] Gómez-Cruz I., Cara C., Romero I., Castro E., Gullón B. (2020). Valorisation of Exhausted Olive Pomace by an Eco-Friendly Solvent Extraction Process of Natural Antioxidants. Antioxidants.

[47] Aissa I., Kharrat N., Aloui F., Sellami M., Bouaziz M., Gargouri Y. (2017). Valorization of antioxidants extracted from olive mill wastewater. Biotechnol. Appl. Biochem..

[48] Belaqziz M., Tan S.P., El-Abbassi A., Kiai H., Hafidi A., O’Donovan O., McLoughlin P. (2017). Assessment of the antioxidant and antibacterial activities of different olive processing wastewaters. PLoS ONE.

[49] Caporaso N., Formisano D., Genovese A. (2018). Use of phenolic compounds from olive mill wastewater as valuable ingredients for functional foods. Crit. Rev. Food Sci. Nutr..

[50] Sar T., Akbas M.Y. (2023). Antimicrobial Activities of Olive Oil Mill Wastewater Extracts against Selected Microorganisms. Sustainability.

[51] Abu-Lafi S., Al-Natsheh M.S., Yaghmoor R., Al-Rimawi F. (2017). Enrichment of Phenolic Compounds from Olive Mill Wastewater and In Vitro Evaluation of Their Antimicrobial Activities. Evid. Based Complement. Altern. Med..

[52] Roila R., Branciari R., Ranucci D., Ortenzi R., Urbani S., Servili M., Valiani A. (2016). Antimicrobial Activity of Olive Mill Wastewater Extract Against *Pseudomonas fluorescens* Isolated from Mozzarella Cheese. Ital. Food Saf..

[53] Sicuro B., Barbera S., Daprà F., Gai F., Gasco L., Paglialonga G., Palmegiano G.B., Vilella S. (2010). The olive oil by-product in ‘rainbow trout *Onchorynchus mykiss* (Walbaum)’ farming: Productive results and quality of the product. Aquac. Res..

[54] Sicuro B., Badino P., Daprà F., Gai F., Galloni M., Odore R., Palmegiano G.B., Macchi E. (2010). Physiological effects of natural olive oil antioxidants utilization in rainbow trout (*Onchorynchus mykiss*) feeding. Aquac. Int..

[55] Sicuro B., Daprà F., Gai F., Palmegiano G.B., Schiavone R., Zilli L., Vilella S. (2010). Olive oil by-product as a natural antioxidant in gilthead sea bream (*Sparus aurata*) nutrition. Aquac. Int..

[56] Hazreen-Nita M.K., Abdul Kari Z., Mat K., Rusli N.D., Mohamad Sukri S.A., Che Harun H., Lee S.W., Rahman M.M., Norazmi-Lokman N.H., Nur-Nazifah M. (2022). Olive oil by-products in aquafeeds: Opportunities and challenges. Aquac. Rep..

[57] Dali I., Aydi A., Stamenic M., Kolsi L., Ghachem K., Zizovic I., Manef A., Delgado D.R. (2022). Extraction of lyophilized olive mill wastewater using supercritical CO_2_ processes. Alex. Eng. J..

[58] Venturi F., Sanmartin C., Taglieri I., Nari A., Andrich G., Terzuoli E., Donnini S., Nicolella C., Zinnai A. (2017). Development of Phenol-Enriched Olive Oil with Phenolic Compounds Extracted from Wastewater Produced by Physical Refining. Nutrients.

[59] Yilmaz S., Sova M., Ergün S. (2018). Antimicrobial activity of trans-cinnamic acid and commonly used antibiotics against important fish pathogens and nonpathogenic isolates. J. Appl. Microbiol..

[60] Rota M.C., Herrera A., Martínez R.M., Sotomayor J.A., Jordán M.J. (2008). Antimicrobial activity and chemical composition of *Thymus vulgaris*, *Thymus zygis* and *Thymus hyemalis* essential oils. Food Control.

[61] CLSI (2020). Clinical and Laboratory Standards Institute. Performance Standards for Antimicrobial Susceptibility Testing of Bacteria Isolated from Aquatic Animals.

[62] Chen J., Sun R., Pan C., Sun Y., Mai B., Li Q.X. (2020). Antibiotics and Food Safety in Aquaculture. J. Agric. Food Chem..

[63] Vincent A.T., Gauthier J., Derome N., Charette S.J., Derome N. (2019). The Rise and Fall of Antibiotics in Aquaculture. Microbial Communities in Aquaculture Ecosystems: Improving Productivity and Sustainability.

[64] Cabello F.C. (2006). Heavy use of prophylactic antibiotics in aquaculture: A growing problem for human and animal health and for the environment. Environ. Microbiol..

[65] Reverter M., Tapissier-Bontemps N., Sasal P., Saulnier D. (2017). Use of Medicinal Plants in Aquaculture. Diagnosis and Control of Diseases of Fish and Shellfish.

[66] Citarasu T. (2010). Herbal biomedicines: A new opportunity for aquaculture industry. Aquac. Int..

[67] Zhu F. (2020). A review on the application of herbal medicines in the disease control of aquatic animals. Aquaculture.

[68] Van Hai N. (2015). The use of medicinal plants as immunostimulants in aquaculture: A review. Aquaculture.

[69] Agoramoorthy G., Chandrasekaran M., Venkatesalu V., Hsu M. (2007). Antibacterial and antifungal activities of fatty acid methyl esters of the blind-your-eye mangrove from India. Braz. J. Microbiol..

[70] McGaw L.J., Jäger A.K., van Staden J. (2002). Isolation of antibacterial fatty acids from Schotia brachypetala. Fitoterapia.

[71] Yu K., Huang Z., Xiao Y., Wang D. (2022). *Shewanella* infection in humans: Epidemiology, clinical features and pathogenicity. Virulence.

[72] Austin B., Austin D.A. (2016). Bacterial Fish Pathogens: Disease of Farmed and Wild Fish.

[73] Hickey M.E., Lee J.-L. (2018). A comprehensive review of *Vibrio (Listonella) anguillarum*: Ecology, pathology and prevention. Rev. Aquac..

[74] Abbott S.L., Kokka R.P., Janda J.M. (1991). Laboratory investigations on the low pathogenic potential of *Plesiomonas shigelloides*. J. Clin. Microbiol..

[75] Brenden R.A., Miller M.A., Janda J.M. (1988). Clinical Disease Spectrum and Pathogenic Factors Associated with *Plesiomonas shigelloides* infections in humans. Rev. Infect. Dis..

[76] Roila R., Primavilla S., Ranucci D., Galarini R., Paoletti F., Altissimi C., Valiani A., Branciari R. (2024). The Effects of Encapsulation on the In Vitro Anti-Clostridial Activity of Olive Mill Wastewater Polyphenolic Extracts: A Promising Strategy to Limit Microbial Growth in Food Systems. Molecules.

[77] Russo E., Spallarossa A., Comite A., Pagliero M., Guida P., Belotti V., Caviglia D., Schito A.M. (2022). Valorization and Potential Antimicrobial Use of Olive Mill Wastewater (OMW) from Italian Olive Oil Production. Antioxidants.

[78] Roila R., Ranucci D., Valiani A., Galarini R., Servili M., Branciari R. (2019). Antimicrobial and anti-biofilm activity of olive oil by-products against *Campylobacter* spp. isolated from chicken meat. Acta Sci. Pol. Technol. Aliment..

[79] Praveen P.K., Debnath C., Shekhar S., Dalai N., Ganguly S. (2016). Incidence of *Aeromonas* spp. infection in fish and chicken meat and its related public health hazards: A review. Vet. World.

[80] Guz L., Kozinska A. (2004). Antibiotic susceptibility of *Aeromonas hydrophila* and *A. sobria* isolated from farmed carp (*Cyprinus carpio* L.). Bull. Vet. Inst. Pulawy.

[81] Cai W.-q., Li S.-f., Ma J.-y. (2004). Diseases resistance of Nile tilapia (*Oreochromis niloticus*), blue tilapia (*Oreochromis aureus*) and their hybrid (female Nile tilapia×male blue tilapia) to *Aeromonas sobria*. Aquaculture.

[82] Ramos-Cormenzana A., Juárez-Jiménez B., Garcia-Pareja M.P. (1996). Antimicrobial activity of olive mill wastewaters (alpechin) and biotransformed olive oil mill wastewater. Int. Biodeterior. Biodegrad..

[83] Yakhlef W., Arhab R., Romero C., Brenes M., de Castro A., Medina E. (2018). Phenolic composition and antimicrobial activity of Algerian olive products and by-products. LWT.

[84] Bisignano G., Tomaino A., Cascio R.L., Crisafi G., Uccella N., Saija A. (1999). On the In-vitro Antimicrobial Activity of Oleuropein and Hydroxytyrosol. J. Pharm. Pharmacol..

[85] Canal C., Ozen B., Baysal A.H. (2019). Characterization of antimicrobial activities of olive phenolics on yeasts using conventional methods and mid-infrared spectroscopy. J. Food Sci. Technol..

[86] Fernandez-Soto P., Celi D., Tejera E., Alvarez-Suarez J.M., Machado A. (2023). *Cinnamomum* sp. and *Pelargonium odoratissimum* as the Main Contributors to the Antibacterial Activity of the Medicinal Drink Horchata: A Study Based on the Antibacterial and Chemical Analysis of 21 Plants. Molecules.

